# Acute Bilateral Stroke in a Moyamoya Patient With High Risk of Thrombosis Due to Multiple Myeloma With Chemotherapy

**DOI:** 10.7759/cureus.34172

**Published:** 2023-01-24

**Authors:** David F Castañeda-Hurtado, Daniela Perez-Samano, Mariana Rios-Gomez, Juan E Montes-Castañeda, Juan E Montes-Ramirez

**Affiliations:** 1 Department of Neurology, Hospital General de México Dr. Eduardo Liceaga, Mexico City, MEX; 2 Department of Hematology, Hospital General de México Dr. Eduardo Liceaga, Mexico City, MEX; 3 Department of Internal Medicine, Hospital Regional de PEMEX en Salamanca, Salamanca, MEX; 4 Department of Neurology, Unidad Médica de Alta Especialidad No. 71 Instituto Mexicano del Seguro Social (IMSS), Torreon, MEX

**Keywords:** arterial thrombosis, venous thromboembolism, moyamoya syndrome, moyamoya disease, multiple myeloma

## Abstract

Multiple myeloma (MM) is a common hematologic malignancy. Multi-agent chemotherapy and anti-myeloma immunomodulatory drugs increase the incidence of arterial and venous thrombosis. We present a moyamoya patient with MM who had a stroke shortly after induction chemotherapy.

We present the case of an adult female patient who arrived at the ER due to automatism seizures, dysarthria, and left hemiparesis. The patient had a medical history of MM and underwent six cycles of induction chemotherapy (cyclophosphamide, dexamethasone, thalidomide, and bortezomib). MRI of the brain showed bilateral watershed ischemic strokes. Angiogram showed occlusion at the supraclinoid segment of both internal carotid arteries consistent with moyamoya. The patient was discharged with full-dose anticoagulation, levetiracetam, and physical therapy. At three years of follow-up, the patient has no recurrent cerebrovascular disease.

MM patients treated with thalidomide/lenalidomide in combination with high-dose dexamethasone, doxorubicin, or multiagent chemotherapy should be on anticoagulation for venous thromboembolism (VTE) prophylaxis. There are no clear recommendations for arterial thrombosis prevention. Moyamoya is a vasculopathy characterized by progressive intracranial artery stenosis with a high risk of ischemic stroke, ischemia recurrence, and intracerebral hemorrhage. Despite the risk of intracerebral hemorrhage, we decided on anticoagulation due to the high risk of thrombosis due to MM, multi-agent chemotherapy, and moyamoya.

## Introduction

Multiple myeloma (MM) accounts for 13% of all hematologic malignancies with an overall 10 years survival of 30% [1]. Multi-agent chemotherapy, anti-myeloma immunomodulatory drugs, and autologous hematopoietic cell transplantation (HCT) have increased survival to more than five years. However, multi-agent chemotherapy and anti-myeloma immunomodulatory drugs increase the incidence of venous thromboembolism (VTE) from 3 to 26%. The risk of arterial thromboembolism (ATE) is also high in these patients [2,3]. We present a challenging case of a patient at high risk of thrombosis from MM who had a stroke shortly after induction chemotherapy with a concurrent high risk of hemorrhage from moyamoya.

## Case presentation

A 42-year-old female patient arrived at the emergency room (ER) due to automatism seizures. At her residence, the patient complained of abdominal pain and had a transient episode of left eye deviation with impaired awareness after which she seemed confused and didn’t remember the episode. Three hours later the patient had dysarthria and left hemiparesis. During sleep, she had an episode of lip-smacking, and left eye deviation with impaired awareness, after which she remained confused. Thus, the patient was brought to the ER. At admission, levetiracetam was given at a dose of 1 g twice daily and complete seizure remission was achieved. The patient had a past medical history of a pathological right femur fracture due to MM diagnosed one year before the seizures. The patient was eligible for autologous hematopoietic cell transplantation and underwent six cycles of induction chemotherapy. The induction chemotherapy included cyclophosphamide, dexamethasone (1200 mg/month), thalidomide, and bortezomib. Despite the high risk of thrombosis from thalidomide and high-dose dexamethasone use thromboprophylaxis with only 100 mg of aspirin daily was given.

**Figure 1:**
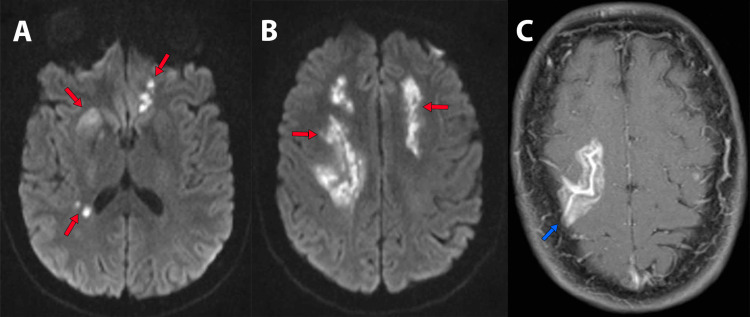
A-B, MRI-DWI shows bilateral white matter hyperintensities at centrum semiovale and high convexity level affecting the ACA-MCA watershed territories (red arrows). C, T1 postcontrast MRI showed leptomeningeal enhancement along the right cortical sulci compatible with an “ivy sign” (blue arrow). DWI: diffusion-weighted imaging, ACA: anterior cerebral artery, MCA: middle cerebral artery

At the ER, the patient’s blood pressure was 110/70 mmHg, her heart rate was 68 bpm, her respiratory rate was 18 bpm, glucose was 103 mg/dL. On examination, the patient was somnolent but arousable to speech, she had right eye deviation, left central facial palsy, left hemiplegia, left hyperreflexia, and a left extensor toe response. Her National Institute of Health stroke score (NIHSS) was 13. Magnetic resonance imaging (MRI) of the brain showed bilateral hyperintensities in the anterior cerebral artery (ACA)-middle cerebral artery (MCA) border zone territories consistent with a bilateral watershed ischemic stroke (Figure 1A, 1B).

**Figure 2:**
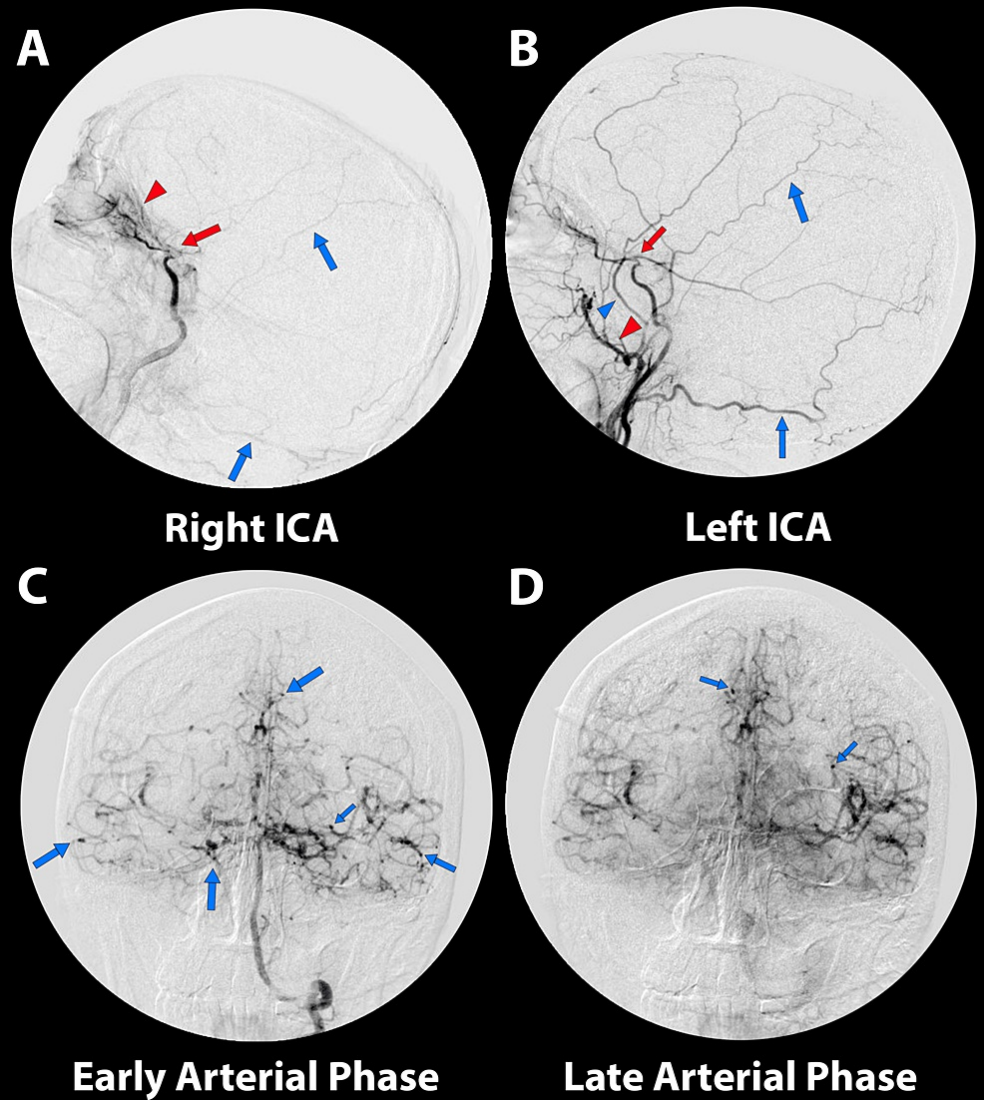
Lateral view right ICA angiogram shows occlusion at the supraclinoid segment of the ICA (A, red arrow), anastomosis of the ophthalmic artery with the posterior ethmoidal artery (A, red arrowhead), and dural anastomosis (A, blue arrows). Lateral view left ICA angiogram shows occlusion at the supraclinoid segment of the ICA (B, red arrow), anastomosis with the temporal artery (B, red arrowhead), middle meningeal artery (B, blue arrowhead), and dural anastomosis (B, blue arrows). Anterior view of left vertebral artery angiogram shows multiple anastomoses (C, blue arrows) and well-developed posterior cerebral arteries which supply the ACA and MCA territories (D). ICA: internal carotid artery, ACA: anterior cerebral artery, MCA: middle cerebral artery

T1 postcontrast MRI showed right leptomeningeal enhancement resembling an “ivy sign” (Figure 1C). An angiogram was requested which showed occlusion at the supraclinoid segment of both internal carotid arteries (ICA) with ethmoidal and dural arteries anastomosis consistent with a bilateral stage V moyamoya. The angiogram of the vertebral arteries showed narrowing of the basilar artery, a well-developed posterior cerebral artery (PCA) supplying the vascular territories of the MCA and ACA, and multiple anastomoses (Figure 2).

The patient’s laboratory results were hemoglobin of 15.5 mg/dl and only relevant for high cholesterol, high low-density lipoprotein (LDL) cholesterol, and high fibrinogen. Her echocardiogram, Holter monitoring, immunologic testing, and cerebrospinal fluid (CSF) were normal.

The patient was discharged to her residence with indefinite full-dose anticoagulation of rivaroxaban, levetiracetam 1 g twice daily, and physical therapy. Consultation for indirect surgical revascularization is pending due to the COVID-19 pandemic. At three years of follow-up, the patient has no recurrent cerebrovascular disease, remains without seizures, has moderate disability able to walk unassisted, and is being treated with lenalidomide and bortezomib monthly achieving minimal residual disease activity from her MM.

## Discussion

The incidence of VTE in MM patients is high despite the current use of agents with less risk of thrombosis such as bortezomib. Thus, thromboprophylaxis is recommended and risk stratification should be assessed for adequate management. The individual risk factors for thromboprophylaxis are obesity, prior VTE, central venous access device, cardiac disease, chronic renal disease, diabetes, acute infection, immobilization, surgery, use of erythropoietin, and blood clotting disorders. The myeloma-related risk factors for thromboprophylaxis are the diagnosis of myeloma itself and hyperviscosity. Myeloma therapy risk factors for thromboprophylaxis are the use of thalidomide or leflunomide in combination with high-dose dexamethasone (≥480 mg per month), doxorubicin, or multiagent chemotherapy. Patients with no or one individual/myeloma-related risk factor should be treated with Aspirin. Patients with two or more individual/myeloma-related risk factors or patients using myeloma therapies with a high risk for thrombosis should be treated with low-molecular-weight heparin (LMWH) or warfarin (target international normalized ratio [INR] 2-3) [2]. Our patient had MM and was treated with thalidomide, multiagent chemotherapy, and high-dose dexamethasone thus she had a high risk for VTE and should have been anticoagulated for VTE prophylaxis.

The risk of ATE is also high in MM patients. A population-based study in Sweden reported an incidence of ATE (acute myocardial infarction, angina, stroke, transient ischemic attack [TIA], peripheral artery embolism, and mesenteric artery thrombosis) of 3.8% in the first year shortly after induction chemotherapy. ATE is a serious complication that increases the risk of death by 3.4 times. It is not known if ATE is a complication from the treatment, if it’s due to the patient’s comorbidities, or the MM itself [3-5]. In the few studies that evaluated ATE in MM patients, family history of premature atherosclerotic cardiovascular disease (ASCVD), race, conditions specific to women (preeclampsia, premature menopause), dyslipidemia, metabolic syndrome, cardiac heart failure, cardiac arrhythmia, symptomatic and asymptomatic arterial stenosis were not considered [2,3,5,6]. Thus, adequate primary and secondary prevention for ATE has not been established in these high-risk patients. In the study of Libourel et al., seven of the 11 ATE occurred despite the use of clopidogrel, prophylactic doses of LMWH, or vitamin K antagonists [6].

Moyamoya is a vasculopathy characterized by progressive intracranial artery stenosis due to vessel wall thickening and the development of small collateral vessels due to angiogenesis. In Hispanics, it’s a rare disease with an incidence of 0.5 per 100,00 persons and a female predominance (1.9:1). It has a bimodal age of presentation at 10 and 40 years of age. The term “moyamoya” refers to the angiographic finding of stenosis or occlusion of intracranial arteries with prominent arterial collateral circulation giving the appearance “like a puff of cigarette smoke”. The fibrocellular intimal thickening seen in moyamoya may also affect other arteries such as carotid, vertebrobasilar, renal, pulmonary, and coronary vessels [7,8].

The most common initial presentation of moyamoya is symptoms of brain ischemia (ischemic stroke and TIA) due to stenosis of the intracranial arteries. In up to 61% of the patients, this initial presentation is ischemic stroke which is also the most common recurrent cerebrovascular event. The recurrence of cerebrovascular events is as high as 55%. Hypoperfusion is also a common cause of watershed strokes in moyamoya patients. Hemorrhage is also frequent especially in adults (10%) due to fragile and dilated collateral vessels [8]. Thus, primary and secondary prevention with antiplatelet agents has been used [9]. Cilostazol has even been shown to increase cerebral blood flow in PET scans and improve cognition in these patients [9]. Anticoagulation is not recommended due to the risk of hemorrhage and difficulty maintaining therapeutic levels [10].

Moyamoya is classified as moyamoya disease (MMD) and moyamoya syndrome (MMS). MMD is reserved for patients who may have genetic susceptibility with no associated condition and is also called primary, idiopathic, or sporadic MMD, while MMS is reserved for patients with an angiographic finding of moyamoya with associated conditions (diseases affecting arteries, hematological conditions, vasculitis, autoimmune diseases, metabolic diseases) and is also called moyamoya phenomenon, angiographic moyamoya, or secondary moyamoya [8].

Our patient seemed to be a case of MMS since there were no hereditary traits, the patient had a recent diagnosis of MM and recently received multiagent chemotherapy which conferred a high risk of thrombosis. But we want to highlight that these conditions have not been previously associated with moyamoya. We didn´t consider hypoperfusion as the cause of watershed strokes since hypotension episodes were never documented.

This is a challenging case with a concurrent risk for thrombosis from MM and chemotherapy, and a high risk of hemorrhage with MMS. Given that our patient was on aspirin when the stroke happened, we chose to anticoagulate indefinitely to prevent further thrombosis.

## Conclusions

This is a unique case of a MM patient with a prothrombotic state from chemotherapy with underlying moyamoya. Moyamoya's high risk of bleeding makes the benefit of vitamin K antagonists unproven. However, the high-risk prothrombotic state of the patient, the ischemic stroke despite antiplatelet use and the improved safety profiles of direct oral anticoagulants made us choose long-term anticoagulation with rivaroxaban.
